# Supplementary material for: HIVprotI: an integrated web based platform for prediction and design of HIV proteins inhibitors
Source: J Cheminform. 2018 Mar 9;10:12. doi: 10.1186/s13321-018-0266-y (PMC5845081; doi:10.1186/s13321-018-0266-y)
Supplement: Supplementary file 2 — Additional file 2. Source code of HIVProtI web server. [file 13321_2018_266_MOESM2_ESM.zip › HIVprotI_Source-code/hivprotI/weka-3-6-6/doc/weka/gui/SimpleCLIPanel.CommandlineCompletion.html]

SimpleCLIPanel.CommandlineCompletion


---


|  |  |  |
| --- | --- | --- |
| **Overview** \| **Package** \| **Class** \| **Tree** \| **Deprecated** \| **Index** \| **Help** \| **Weka's home** | |  |
| **PREV CLASS**   **NEXT CLASS** | **FRAMES**    **NO FRAMES**     **All Classes** |  |
| SUMMARY: NESTED \| FIELD \| CONSTR \| METHOD | DETAIL: FIELD \| CONSTR \| METHOD |  |


---


## weka.gui Class SimpleCLIPanel.CommandlineCompletion

```
java.lang.Object
  weka.gui.SimpleCLIPanel.CommandlineCompletion
```

**Enclosing class:**: SimpleCLIPanel

---

``` public static class SimpleCLIPanel.CommandlineCompletion extends java.lang.Object ```

A class for commandline completion of classnames.

**Version:**
:   $Revision: 7059 $

**Author:**
:   FracPete (fracpete at waikato dot ac dot nz)

---

**Constructor Summary**

`SimpleCLIPanel.CommandlineCompletion()`
  
          default constructor.


| **Method Summary** | |
| --- | --- |
| `java.util.Vector<java.lang.String>` | `getClassMatches(java.lang.String partial)`             returns all the class/package matches with the partial search string. |
| `java.lang.String` | `getClassname(java.lang.String partial)`             returns the classname part of the partial classname. |
| `java.lang.String` | `getCommonPrefix(java.util.Vector<java.lang.String> list)`             returns the common prefix for all the items in the list. |
| `boolean` | `getDebug()`             returns whether debug mode is on. |
| `java.util.Vector<java.lang.String>` | `getFileMatches(java.lang.String partial)`             returns all the file/dir matches with the partial search string. |
| `java.util.Vector<java.lang.String>` | `getMatches(java.lang.String partial)`             returns all the matches with the partial search string, files or classes. |
| `java.lang.String` | `getPackage(java.lang.String partial)`             returns the packages part of the partial classname. |
| `boolean` | `isClassname(java.lang.String partial)`             tests whether the given partial string is the name of a class with classpath - it basically tests, whether the string consists only of alphanumeric literals, underscores and dots. |
| `void` | `setDebug(boolean value)`             sets debug mode on/off. |

**Methods inherited from class java.lang.Object**

`equals, getClass, hashCode, notify, notifyAll, toString, wait, wait, wait`

**Constructor Detail**

### SimpleCLIPanel.CommandlineCompletion

```
public SimpleCLIPanel.CommandlineCompletion()
```

:   default constructor.


**Method Detail**

### getDebug

```
public boolean getDebug()
```

:   returns whether debug mode is on.

    :   **Returns:**: true if debug is on

---


### setDebug

```
public void setDebug(boolean value)
```

:   sets debug mode on/off.

    :   **Parameters:**: `value` - if true then debug mode is on

---


### isClassname

```
public boolean isClassname(java.lang.String partial)
```

:   tests whether the given partial string is the name of a class with
    classpath - it basically tests, whether the string consists only
    of alphanumeric literals, underscores and dots.

    :   **Parameters:**: `partial` - the string to test **Returns:**: true if it looks like a classname

---


### getPackage

```
public java.lang.String getPackage(java.lang.String partial)
```

:   returns the packages part of the partial classname.

    :   **Parameters:**: `partial` - the partial classname **Returns:**: the package part of the partial classname

---


### getClassname

```
public java.lang.String getClassname(java.lang.String partial)
```

:   returns the classname part of the partial classname.

    :   **Parameters:**: `partial` - the partial classname **Returns:**: the class part of the classname

---


### getFileMatches

```
public java.util.Vector<java.lang.String> getFileMatches(java.lang.String partial)
```

:   returns all the file/dir matches with the partial search string.

    :   **Parameters:**: `partial` - the partial search string **Returns:**: all the matches

---


### getClassMatches

```
public java.util.Vector<java.lang.String> getClassMatches(java.lang.String partial)
```

:   returns all the class/package matches with the partial search string.

    :   **Parameters:**: `partial` - the partial search string **Returns:**: all the matches

---


### getMatches

```
public java.util.Vector<java.lang.String> getMatches(java.lang.String partial)
```

:   returns all the matches with the partial search string, files or
    classes.

    :   **Parameters:**: `partial` - the partial search string **Returns:**: all the matches

---


### getCommonPrefix

```
public java.lang.String getCommonPrefix(java.util.Vector<java.lang.String> list)
```

:   returns the common prefix for all the items in the list.

    :   **Parameters:**: `list` - the list to return the common prefix for **Returns:**: the common prefix of all the items


---


|  |  |  |
| --- | --- | --- |
| **Overview** \| **Package** \| **Class** \| **Tree** \| **Deprecated** \| **Index** \| **Help** \| **Weka's home** | |  |
| **PREV CLASS**   **NEXT CLASS** | **FRAMES**    **NO FRAMES**     **All Classes** |  |
| SUMMARY: NESTED \| FIELD \| CONSTR \| METHOD | DETAIL: FIELD \| CONSTR \| METHOD |  |


---
